# Glucose-6-phosphate 1-Epimerase CrGlu6 Contributes to Development and Biocontrol Efficiency in *Clonostachys chloroleuca*

**DOI:** 10.3390/jof9070764

**Published:** 2023-07-20

**Authors:** Binna Lv, Yan Guo, Xue Zhao, Shidong Li, Manhong Sun

**Affiliations:** Institute of Plant Protection, Chinese Academy of Agricultural Sciences, Beijing 100193, China; lvbinna03@163.com (B.L.); zhaoxue20200220@163.com (X.Z.); sdli@ippcaas.cn (S.L.)

**Keywords:** biocontrol, *Clonostachys chloroleuca*, glucose-6-phosphate 1-epimerase, MAPK, mycoparasitism, *Sclerotinia sclerotiorum*

## Abstract

*Clonostachys chloroleuca* (formerly classified as *C. rosea*) is an important mycoparasite active against various plant fungal pathogens. Mitogen-activated protein kinase (MAPK) signaling pathways are vital in mycoparasitic interactions; they participate in responses to diverse stresses and mediate fungal development. In previous studies, the MAPK-encoding gene *Crmapk* has been proven to be involved in mycoparasitism and the biocontrol processes of *C. chloroleuca*, but its regulatory mechanisms remain unclear. Aldose 1-epimerases are key enzymes in filamentous fungi that generate energy for fungal growth and development. By protein–protein interaction assays, the glucose-6-phosphate 1-epimerase CrGlu6 was found to interact with Crmapk, and expression of the *CrGlu6* gene was significantly upregulated when *C. chloroleuca* colonized *Sclerotinia sclerotiorum* sclerotia. Gene deletion and complementation analyses showed that *CrGlu6* deficiency caused abnormal morphology of hyphae and cells, and greatly reduced conidiation. Moreover, deletion mutants presented much lower antifungal activities and mycoparasitic ability, and control efficiency against sclerotinia stem rot was markedly decreased. When the *CrGlu6* gene was reinserted, all biological characteristics and biocontrol activities were recovered. These findings provide new insight into the mechanisms of glucose-6-phosphate 1-epimerase in mycoparasitism and help to further reveal the regulation of MAPK and its interacting proteins in the biocontrol of *C. chloroleuca*.

## 1. Introduction

*Clonostachys rosea* is a promising mycoparasite capable of attacking a range of plant pathogenic fungi including *Sclerotinia sclerotiorum*, *Fusarium oxysporum*, and *Rhizoctonia solani* [1,2,3,4]. The biocontrol mechanisms of this species mainly contain mycoparasitism, competition, secretion of cell wall-degrading enzymes, and production of antimicrobial compounds and stimulants such as peptaibols, polyketides, and indole alkaloids [5,6,7,8]. Functional genes of *C. rosea* known to be linked to mycoparasitism and biocontrol actions include chitinase gene *chiC2*, pectate lyase gene *pel12*, and heat shock protein 70 gene *crhsp* [9,10,11]. Based on a recent phylogenetic study [12], *Clonostachys chloroleuca* was characterized as a separate species. So far, *C. chloroleuca* has only been reported from a collection of soilborne isolates from Brazil, and reports on the biocontrol potential of this species are scarce in the literature [12,13]. However, strain 67-1, formerly identified as *C. rosea* and used as a biocontrol agent against *Fusarium* and *Sclerotinia*-incited diseases in our previous studies [14,15], was found to belong to *C. chloroleuca* [12]. Meanwhile, it has been proven that mycoparasitism and biocontrol potential are traits shared by different species within the genus and are not exclusive to *C. rosea* [16].

In mycoparasitism, a living fungus is colonized by, and acts as a nutrient source for, another fungus [17,18,19]. During fungal interactions, mycoparasites recognize molecular signals released by host fungi through several signaling pathways [17,20]. Among these pathways, mitogen-activated protein kinases (MAPKs) are key mediators of signaling, participating in responses to diverse stresses and fungal development [21,22,23]. A typical MAPK pathway comprises a cascade of three consecutive phosphorylation events exerted by serine/threonine kinases known as MAPK kinase kinase (MAPKKK or MAP3K), MAPK kinase (MAPKK or MAP2K), and MAPK. Activated MAPKs induce specific physiological responses to initiate exogenous signals by phosphorylating downstream target proteins and transcription factors [24,25,26].

MAPK pathways have been well studied in the yeast *Saccharomyces cerevisiae* as a model organism, from which five MAPKs, Fus3, Kss1, Hog1, Slt2, and Smk1, were found to regulate pheromone responses, filamentation and invasiveness, high osmolarity growth, cell wall integrity, and ascospore cell assembly, respectively [27,28,29]. In pathogenic filamentous fungi, only three MAPKs (orthologous to yeast Fus3/Kss1, Slt2, and Hog1) have been reported, and they display various functions related to pathogenesis, differentiation, and stress responses [30,31]. In mycoparasites, MAPKs are also widely distributed and involved in multiple biological and biocontrol processes. When MAPK-encoding genes were deficient, the parasitic abilities of *Trichoderma virens* (*Tmk1* and *Tvk1*), *Coniothyrium minitans* (*CmSlt2*), and *C. chloroleuca* (*Crmapk*) were affected [32,33,34,35]. However, molecular mechanisms by which MAPKs regulate mycoparasitism remain unclear, as do the downstream receptors.

During signal transmission, a series of proteins interacting with MAPKs are activated and work cooperatively to respond to external stimuli. MAPKs can interact with various proteins in plant pathogenic fungi. In *Magnaporthe grisea*, the *MST12* gene homologous to the transcription factor gene *Ste12* in *S. cerevisiae* encodes a vital transcription factor that functions downstream of MAP kinase PMK1 to regulate genes involved in appressoria penetration and growth of infectious hyphae [36]. Similar findings were also reported in *Fusarium oxysporum*, showing that Ste12 controlled invasive growth and pathogen virulence by acting downstream of the MAP kinase Fmk1 [37]. In *C. chloroleuca*, proteins potentially interacting with Crmapk (an ortholog of *S. cerevisiae Fus3/Kss1*) were screened from a yeast two-hybrid (Y2H) library, most of which were related to metabolism, cellular processes, and signal transduction [38]. Among the interacting proteins, a glucose-6-phosphate 1-epimerase named CrGlu6 was linked to the mechanism by which Crmapk regulates mycoparasitism of *C. chloroleuca*.

Aldose 1-epimerases (mutarotases) are key enzymes of carbohydrate metabolism that catalyze the interconversion of the alpha- and beta-anomers of hexose sugars such as glucose and galactose. In filamentous fungi, these interconversions are of great significance for providing energy for the growth and development of microorganisms [39]. Several aldose 1-epimerases have been identified in fungi that perform various functions. In *T. harzianum*, aldose 1-epimerases were upregulated during interactions with plants [40]. Likewise, homologues in *M. grisea* were differentially expressed during appressorium formation [41]. In *Hypocrea jecorina*, an aldose 1-epimerase was proven to be involved in galactose metabolism, via which the production of cellulase was affected [42]. Among aldose 1-epimerases, glucose-6-phosphate 1-epimerase catalyzes the conversion of alpha- and beta-anomers of hexose 6-phosphate sugars of Glc6P, Gal6P, and Man6P [43], and mainly participates in carbohydrate metabolic processes including glycolysis and gluconeogenesis [44]. Glucose homeostasis is regulated by signal transduction, especially protein kinase signaling [44], but the roles of glucose-6-phosphate 1-epimerase and its relationship with MAPK pathways in *C. chloroleuca* during the mycoparasitic process have not been elucidated.

In the present study, the glucose-6-phosphate 1-epimerase CrGlu6 was identified from a yeast 2-hybrid (Y2H) library of Crmapk-interacting proteins in *C. chloroleuca* 67-1. The interaction was verified by protein–protein interaction assays, and the results indicated that CrGlu6 might associate with MAPK pathways linked to mycoparasitism of *C. chloroleuca*. The functions of CrGlu6 in *C. chloroleuca* were also investigated, and the protein was found to be involved in fungal growth, conidiation, and biocontrol activities. The findings provide new insight into the mechanisms of glucose-6-phosphate 1-epimerase involved in mycoparasitism and facilitate understanding of the regulation of MAPK and its interacting proteins in the biocontrol of *C. chloroleuca* against *S. sclerotiorum*, the agent of stem rot of soybean [45].

## 2. Materials and Methods

### 2.1. Fungal Strains and Culture Conditions

The wild type *Clonostachys chloroleuca* strain 67-1 (ACCC 39160) and its derived transformants were cultivated and maintained on potato dextrose agar (PDA) under a previously described method [46]. *Sclerotinia sclerotiorum* Ss-H (ACCC 39161) was stored at −80 °C in 30% glycerol and cultured routinely at 26 °C on PDA.

### 2.2. Yeast Two-Hybrid Assay

In a previous study, we found that the glucose-6-phosphate 1-epimerase CrGlu6 potentially interacts with Crmapk in *C. chloroleuca* when mycoparasitizing *S. sclerotiorum* [38]. To verify this interaction, the coding sequence (CDS) of *Crmapk* and *CrGlu6* genes was acquired with specific primers designed by Primer3 (https://www.yeastgenome.org/primer3, accessed on 15 July 2021; Table S1). The resulting PCR fragments were inserted into bait vector pGBKT7 (Clontech, Mountain View, CA, USA) and prey vector pGADT7 (Clontech), separately. After verification by double restriction enzyme digestion and sequencing, the recombinant pGBKT7-Crmapk and pGADT7-CrGlu6 plasmids were co-transformed into the *S. cerevisiae* strain Y2H Gold following the PEG/LiAc-mediated transformation protocol [47]. Plasmids pGBKT7-53 and pGADT7 co-transformed served as positive controls, while vectors pGBKT7-Lam and pGADT7 served as negative controls. Transformants were grown on synthetic medium SD lacking Trp and Leu (DDO) at 30 °C for 3−5 days, then transferred to SD plates lacking His, Trp, Leu, and Ade (QDO) to confirm the interaction of Crmapk and CrGlu6. Three independent experiments were carried out.

### 2.3. Bimolecular Fluorescence Complementation (BiFC) Assay

The *Crmapk* gene with its native promoter was cloned into the pHZ65 plasmid to construct the recombinant YFP^N^-Crmapk vector, which harbored YFP^N^ and the hygromycin B resistance cassette. Likewise, the CrGlu6-YFP^C^ vector was recombined based on the pHZ68 plasmid, which carried YFP^C^ and the zeocin resistance cassette [47]. Both of the two vectors were then transformed into protoplasts of *C. chloroleuca* 67-1 and screened by resistance and PCR. In the meantime, YFP^N^-Crmapk and CrGlu6-YFP^C^ vectors were separately transformed into 67-1 to serve as negative controls. Yellow fluorescent protein (YFP) signals in mycelia cultures on potato dextrose broth (PDB) for 48 h were observed using an LSM980 confocal fluorescence laser-scanning microscope (Zeiss, Gottingen, Germany).

### 2.4. Glutathione S-Transferase (GST) Pull-Down Assay

DNA fragments of Crmapk and CrGlu6 were incorporated into the pGEX-4T-1 vector (GE Healthcare, Chicago, IL, USA) and pCZN1 vector (Zoonbio, Nanjing, China) separately to generate the Crmapk-GST and CrGlu6-His fusion protein. Crmapk-GST, CrGlu6-His, and GST plasmids were transformed into *Escherichia coli* BL21 cells (Sangon, Shanghai, China). Cells were lysed in lysis buffer (50 mM Tris pH 8.0, 50 mM NaCl, 1 mM phenylmethanesulfonyl fluoride [PMSF]) using an ultrasonic processor (Scientz, Ningbo, China) and collected under a high-speed centrifugation. GST and Crmapk-GST supernatants were mixed with 30 μL Glutathione Sepharose beads (GE Healthcare) and incubated at 4 °C for 2 h, and recombinant Crmapk-GST and GST bound to beads were incubated with the *E. coli* cell lysate containing CrGlu6-His at 4 °C. After 4 h, the beads were washed five times with buffer (50 mM Tris pH 8.0, 50 mM NaCl, 1 mM PMSF, 1% Triton X-100), and the eluted proteins were analyzed by immunoblotting with monoclonal anti-His and monoclonal anti-GST antibodies (Abcam, Cambridge, UK) [48].

### 2.5. Bioinformatics Analysis of CrGlu6

The DNA sequence of *CrGlu6* was acquired based on the draft genome sequence of *C. chloroleuca* 67-1 [15]. BLASTp analyses were based on NCBI (http://www.ncbi.nlm.nih.gov/, accessed on 8 June 2021) and UniProt (http://www.uniprot.org/blast/, accessed on 12 June 2021). Conserved functional domains of CrGlu6 were assessed by SMART (http://smart.embl.de/, accessed on 6 July 2021). Amino acid alignments were analyzed using Clustal X program [49]. MEGA 7.0 [50] was used for the construction of the phylogenetic tress following the maximum likelihood method with 1000 bootstrap replicates.

### 2.6. Quantitative Reverse-Transcription PCR (qRT-PCR) Analysis of CrGlu6

Total RNA of *C. chloroleuca* 67-1 mycoparasitizing *S. sclerotiorum* sclerotia was extracted at different stages (8 h, 24 h, and 48 h) using TRIzol reagent (Invitrogen, Carlsbad, CA, USA) following the standard methods. Gene expression of *CrGlu6* in *C. chloroleuca* was monitored by qRT-PCR using a Bio-Rad IQ 5 Real-Time System (Bio-Rad, Hercules, CA, USA). Primers (CrGlu6-F/CrGlu6-R) were designed using Primer3 (Table S1) and the elongation factor gene *EF1* (GenBank accession no. KP274074) served as an internal reference gene to normalize gene expression in the parasitic process [4]. The mycelial samples of *C. chloroleuca* 67-1 without induction of sclerotia served as controls. Relative expression levels of *CrGlu6* were calculated using the 2^−∆∆Ct^ method [51], and three replicates were conducted for each sample.

### 2.7. Gene Deletion and Complementation

The *CrGlu6* gene knockout vector was constructed using the pKH-KO plasmid containing two uracil-specific excision reagent (USER) cloning sites (USC1 and USC2) on either side of the hygromycin resistance gene *hph* [52]. The 5′ and 3′ regions of *CrGlu6* were amplified using primer pairs CrGlu6-UF/CrGlu6-UR and CrGlu6-DF/CrGlu6-DR, separately, and incorporated into two USC sites using the USER-friendly cloning method.

For complementation, the full-length sequence of *CrGlu6* was obtained using 67-1 DNA as a template and recombined into the pKN plasmid, which carried the G418 resistance gene *neo* [53]. The *CrGlu6* gene deletion and complementation vectors obtained above were transformed into the protoplasts of 67-1 and ΔCrGlu6 severally, with the protoplast formation and transformation methods of *C. rosea* [54]. Gene-deficient and complementary mutants were confirmed by PCR assays and DNA sequencing. To further verify the mutants, the expression levels of *CrGlu6* in different strains were determined using RT-PCR with specific primers CrGlu6-F/CrGlu6-R and reference gene *EF-1* (Table S1).

### 2.8. Growth and Conidiation of CrGlu6 Mutants

Phenotypes of *C. chloroleuca* 67-1, ΔCrGlu6, and ΔCrGlu6-C strains were characterized on plates. Agar blocks (3 mm diameter) of the strains cultured on PDA for 2 days were cut from the edges of the colonies and inoculated on the centers of PDA plates. The isolates were grown at 26 °C, and colony expansion was measured daily. After 15 days, the yielded spores were washed with sterile distilled water and counted under a BX41 microscope (Olympus, Tokyo, Japan) with a hemocytometer. Each experiment was repeated three times.

### 2.9. Cellular Morphology of CrGlu6 Mutants

The ultrastructure of *C. chloroleuca* isolates was observed using TEM. Conidia of the wild type, ΔCrGlu6, and ΔCrGlu6-C strains were collected and transferred into a PDB medium and cultured at 26 °C overnight with shaking at 180 rpm. Fresh mycelia were collected, washed, and fixed with 2.5% (*v*/*v*) glutaraldehyde in 0.1 M phosphate-buffered saline (PBS, pH 7.4) at 4 °C for 24 h. The samples were washed three times with the PBS buffer prior to fixing in 1% osmium tetroxide buffered with 0.1 M cacodylate (pH 7.0) at 4 °C overnight, dehydrated in a graded series of ethanol, and infiltrated with a series of epoxy resin in epoxy propane prior to embedding in Epon-812 resin. Ultrathin sections of each sample were cut with an EM UC6 ultramicrotome (Leica, Wetzlar, Germany) and observed under an H-7500 transmission electron microscope (Hitachi, Tokyo, Japan) operating at 80 kV.

### 2.10. Antifungal Activities

To test the antagonistic activities of the wild type strain and mutants of *C. chloroleuca* against *S. sclerotiorum*, a 3-mm agar plug of each strain was placed 2 cm from the left edge of the plate and grown at 26 °C for 5 days. A plug of *S. sclerotiorum* was then placed equidistantly from the right side. After confrontation culture for 3 weeks, the distance of hyphal extension onto the colony of the pathogenic fungus was measured and antifungal activities of the mutants were calculated compared with the wild type [9,55,56].

### 2.11. Mycoparasitic Ability against S. sclerotiorum Sclerotia

Uniform sclerotia were selected and sterilized under 1% NaClO for 3 min, then washed with sterile water several times. Sclerotia were immersed in spore suspensions of different strains at a concentration of 1 × 10^7^ spores/mL for 10 min, then placed onto a piece of wet sterile filter paper in a Petri dish (9 cm diameter) and cultured at 26 °C. Treatment with sterile water was used as a control. One week later, the parasitic severity of sclerotia was counted under a BX41 inverted microscope (Olympus) based on a 4-grade scale as described previously [57]. A total of 30 sclerotia were tested for each treatment with three replicates.

### 2.12. Control Efficacy against Sclerotinia Stem Rot in Greenhouse Experiments

The control effects of *CrGlu6* on sclerotinia stem rot were tested in pots in a greenhouse, in which the susceptible cultivar Zhonghuang 13 (Institute of Crop Sciences, Chinese Academy of Agricultural Sciences, Beijing, China) was employed. The soybean seeds were surface sterilized with 1% NaClO for 3 min, washed thoroughly with tap water, then sown in soil in plastic pots (11 cm diameter). When nine compound leaves had grown, seedlings were inoculated with 100 mL spore suspension (1 × 10^7^ spores/mL) from the wild type, ΔCrGlu6, and ΔCrGlu6-C strains. Two hours later, all leaves were sprayed with an equivalent amount of *S. sclerotiorum* mycelial suspension. Treatments with sterile water were followed by the pathogen as controls, and 15 pots were tested for each strain. After 7 days, disease severity of sclerotinia stem rot was scored and the disease index and control efficiency were assessed [57]. All unfolded compound leaves were checked for each treatment with three replicates.

### 2.13. Statistical Analysis

SPSS 2.0 statistical software (SPSS Inc., Chicago, IL, USA) was used for analysis of variance (ANOVA). Statistical tests were carried out for multiple comparisons using Tukey’s test and *p* < 0.05 was considered statistically significant.

## 3. Results

### 3.1. CrGlu6 Interacts with Crmapk in C. chloroleuca

**Figure 1:**
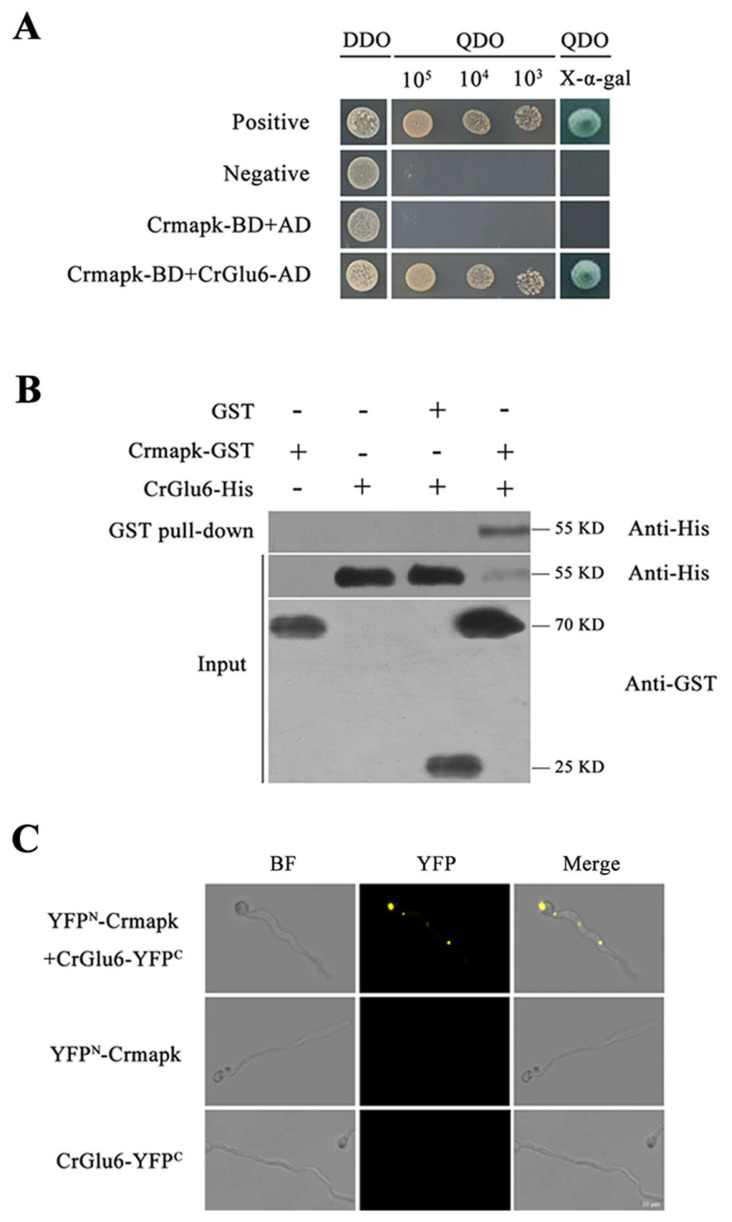
Verification of interaction between CrGlu6 and Crmapk. (**A**) Y2H assay. Yeast transformants expressing bait and prey vectors were incubated on SD−Leu−Trp (DDO) plates and assayed on SD−Ade−His−Leu−Trp (QDO) plates with X-α-gal. (**B**) GST pull-down assay in vitro. Proteins were pulled down using glutathione sepharose beads and eluted samples were analyzed using western blotting with anti-His and anti-GST antibodies. (**C**) BiFC assay in vivo. Transformants co-expressing YFP^N^-Crmapk and CrGlu6-YFP^C^ were observed under a confocal fluorescence microscope. Bar = 10 μm.

Y2H assays of Crmapk-interacting proteins in *C. chloroleuca* 67-1 indicated that CrGlu6 interacted specifically with Crmapk (Figure 1A). This interaction was confirmed by the GST pull-down method, in which CrGlu6-His protein was pulled down using glutathione sepharose beads, yielding an appropriate-sized band by western blotting assay with anti-His antibody. Analyses of input samples using anti-GST or anti-His as a reference resulted in bands of the predicted sizes (Figure 1B). Furthermore, BiFC assays confirmed the interaction between Crmapk and CrGlu6 in vivo; transformants co-expressing YFP^N^-Crmapk and CrGlu6-YFP^C^ exhibited strong YFP signals in the nucleus, whereas no YFP signal was observed in cells transformed with YFP^N^-Crmapk or CrGlu6-YFP^C^ (Figure 1C). These results indicated that the glucose-6-phosphate 1-epimerase CrGlu6 in *C. chloroleuca* interacted with Crmapk in vitro and in vivo.

### 3.2. Identification of CrGlu6 in C. chloroleuca

**Figure 2:**
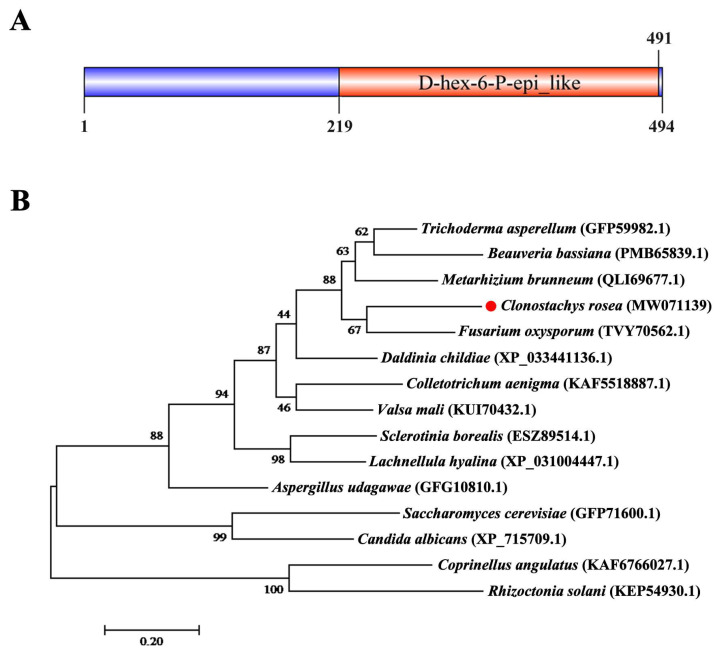
Characterization of CrGlu6 in *C. chloroleuca* 67-1. (**A**) Domain structure of CrGlu6 predicted by SMART MODE (http://smart.embl.de/, accessed on 3 March 2022). (**B**) Phylogenetic analysis of CrGlu6 and its homologs in other fungi. Amino acid sequences were aligned using Clustal X and analyzed using MEGA 7.0 by the maximum likelihood method. Identifications in parentheses represent GenBank accession numbers, and numbers at nodes express the bootstrap values of 1000 repeats. Bars indicate sequence divergence (bar = 0.20).

**Figure 3:**
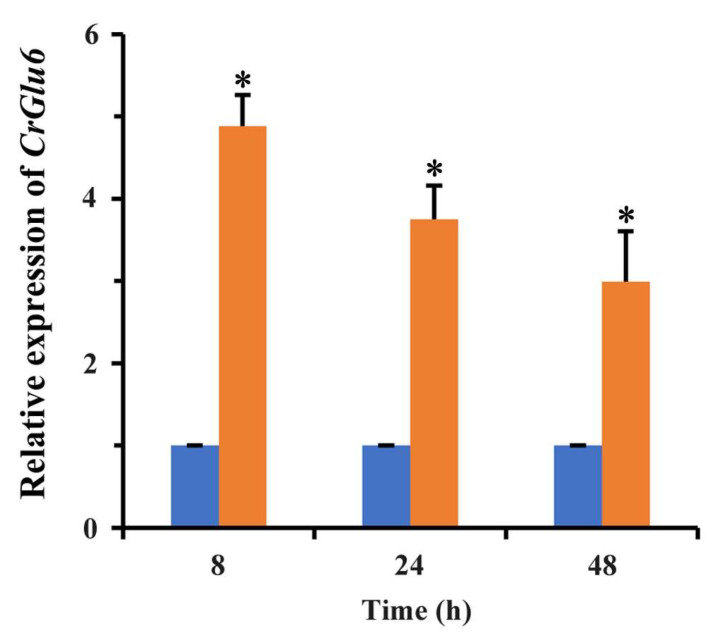
Expression levels of *CrGlu6* in *C. chloroleuca* under sclerotia mycoparasitism conditions. Orange columns indicate *C. chloroleuca* samples treated with fresh sclerotia, while blue columns are controls without sclerotia. Relative expression levels of *CrGlu6* were calculated using the 2^−∆∆Ct^ method. Error bars represent the standard deviations of three replicates. Statistical analyses were performed by Tukey’s test for multiple comparisons, and asterisks represent significant differences (*p* < 0.05).

Gene cloning and bioinformatics analysis showed that *CrGlu6* (GenBank accession number: MW071139) is 1482 bp in length, with three introns, and encodes a 493-amino acid polypeptide that contains a D-hex-6-P-epi_like domain (Figure 2A). It belongs to the aldose-1-epimerase superfamily and participates in carbohydrate metabolism. Phylogenetic analysis and sequence alignment of *CrGlu6* with other fungal species revealed a close relatedness to its homolog in *F. oxysporum* and high conservation among various fungi (Figure 2B). qRT-PCR analysis of gene expression during the mycoparasitic process showed that *CrGlu6* was significantly upregulated (|Log_2_ FC| ≥ 1 and *p* ≤ 0.05) in *C. chloroleuca* throughout mycoparasitism, and expression levels were 4.88, 3.75, and 2.99-fold higher than controls at 8 h, 24 h, and 48 h, respectively (Figure 3), indicating that the *CrGlu6* gene might be of great importance during *C. chloroleuca* parasitizing *S. sclerotiorum*.

### 3.3. Deletion and Complementation of CrGlu6

**Figure 4:**
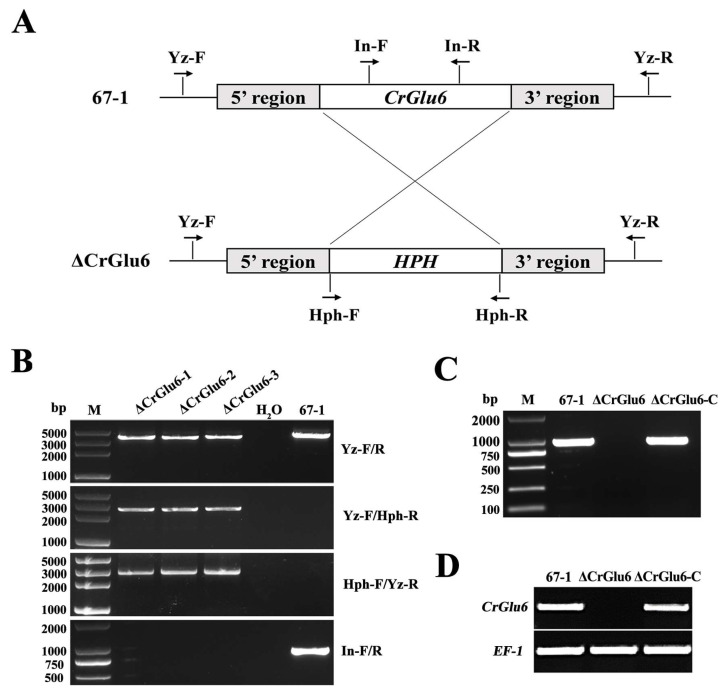
Targeted gene knockout of *CrGlu6* in *C. chloroleuca*. (**A**) Schematic diagram of the gene deletion strategy for *CrGlu6* in *C. chloroleuca* using the homologous recombination method. (**B**) PCR verification of *C. chloroleuca* 67-1 strain and gene deletion mutants with primers Yz-F/R, Yz-F/Hph-R, Hph-F/Yz-R, and In-F/R, severally. The *C. chloroleuca* wild type strain 67-1 was used as a positive control and H_2_O was used as a negative control; M represents DNA molecular markers. “Yz” represents whether the *hph* gene is correctly embedded; “In” represents the *CrGlu6* gene. (**C**) PCR confirmation of the ΔCrGlu6-C strain. *CrGlu6* genes with the same size were detected in the wild type and ΔCrGlu6-C strains but not in the ΔCrGlu6 strain. (**D**) RT-PCR verification of *CrGlu6* gene expressions in 67-1, ΔCrGlu6, and ΔCrGlu6-C strains with specific primers CrGlu6-F/R (Table S1). The RT-PCR product of 283 bp presented in 67-1 and ΔCrGlu6-C while not in the CrGlu6 deficiency mutants.

To investigate the potential functions of *CrGlu6*, a homologous recombination strategy was used to knock out the *CrGlu6* gene in *C. chloroleuca* 67-1 (Figure 4A), and finally three deletion mutants ΔCrGlu6 were generated from 85 hygromycin-resistant transformants verified by PCR products using the primers listed in Table 1 (Figure 4B). The fragments amplified by the primer pair CrGlu6-Yz-F/R were sequenced, indicating that the *CrGlu6* gene was successfully replaced with a hygromycin B resistance cassette, as expected.

For complementation, the full-length *CrGlu6* gene, including the native promoter and terminator region sequences, was transformed into the ΔCrGlu6 strain to generate ΔCrGlu6-C complementation strains. The transformants were confirmed by PCR, and the results demonstrated that specific bands were detected for the wild type and complementation strains, but not for the ΔCrGlu6 mutants (Figure 4C). Finally, 7 complementation strains were identified from 52 transformants, and they all showed a similar appearance to the wild type strain. Furthermore, RT-PCR verification demonstrated a complete loss of *CrGlu6* transcript in ΔCrGlu6 mutants, whereas specific products were detected in the wild type and complementation strains (Figure 4D). In addition, the expression of *EF-1* gene was detected in all strains. These results suggested that the knockout of *CrGlu6* was efficient, and the mutants could be used for further morphological and biological analyses.

### 3.4. Characterization of CrGlu6 in Growth and Conidiation

**Figure 5:**
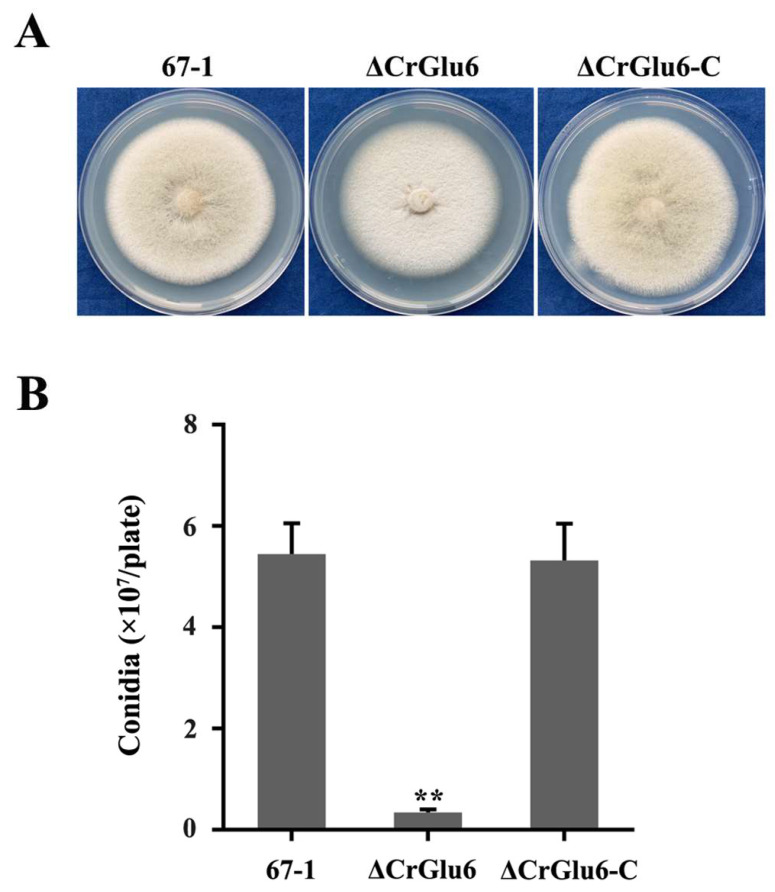
Effects of *CrGlu6* on growth and conidiation of *C. chloroleuca* 67-1, ΔCrGlu6, and ΔCrGlu6-C strains. (**A**) Mycelial growth on PDA medium after 9 days of incubation. (**B**) Conidia production on PDA plates after 15 days. Results are means of the three ΔCrGlu6 and ΔCrGlu6-C mutants, and the means and standard errors were calculated from three independent replicates. Statistical analyses were performed by Tukey’s test for multiple comparisons, and the asterisks represent significant differences (*p* < 0.05).

Three ΔCrGlu6 and ΔCrGlu6-C mutants with identical morphology were selected to explore the roles of *CrGlu6* in the biological processes of *C. chloroleuca* 67-1. Compared with the wild type strain 67-1, the ΔCrGlu6 mutants had flatter colony morphology and reduced conidiation (Figure 5A), despite a normal growth rate (Figure S1). After incubation on PDA for 15 days, only 3.4 × 10^6^ spores/plate were harvested for ΔCrGlu6, compared with 5.5 × 10^7^ and 5.3 × 10^7^ spores/plate for the wild type and complementary strains (*p* < 0.01; Figure 5B).

**Figure 6:**
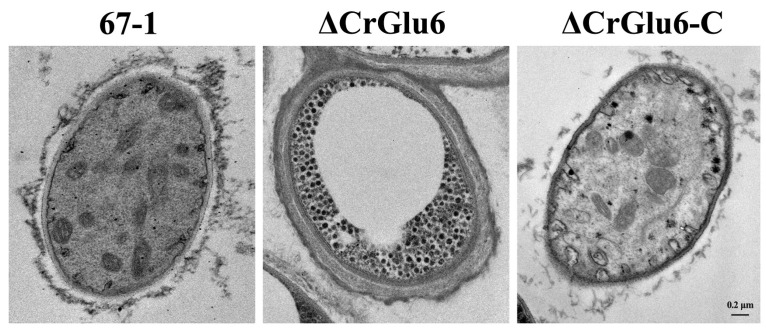
Impacts of *CrGlu6* on the cellular morphology of *C. chloroleuca* hyphae. Ultrastructure was observed under a transmission electron microscope. Cells of 67-1 were filled with organized cytoplasm, mitochondria, and other organelles. By contrast, cells of the ΔCrGlu6 mutants were filled with far fewer organelles and increased glycogen content. Bar = 0.2 μm.

The effects of the *CrGlu6* gene on the cellular morphology of *C. chloroleuca* hyphae were investigated using TEM, and the results showed that *CrGlu6* deficiency caused abnormal ultrastructure of the fungus. The wild type cells had intact cell walls and plasma membranes, organized cytoplasm and mitochondria with well-defined envelopes, and other organelles were also of normal appearance. By contrast, deletion of *CrGlu6* had remarkable effects on cellular morphology, with far fewer organelles and increased glycogen content (Figure 6), suggesting that the *CrGlu6* gene is involved in hyphal functions and activities of *C. chloroleuca*. In the complemented transformants ΔCrGlu6-C, cell morphology was similar to that of the wild type strain.

### 3.5. Characterization of CrGlu6 in Antifungal Activities

**Figure 7:**
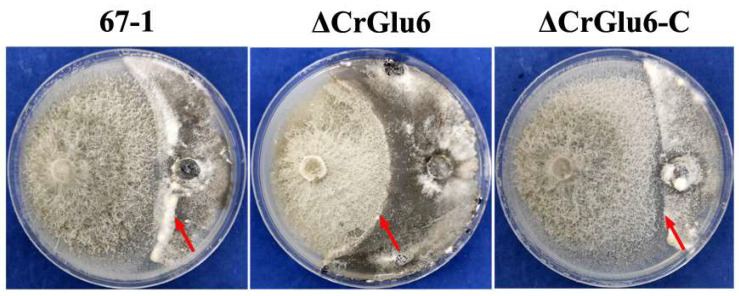
Impacts of *CrGlu6* on the antagonistic activities of *C. chloroleuca* strains. Confrontation cultures of 67-1, ΔCrGlu6, and ΔCrGlu6-*C* against *S. sclerotiorum* were assayed at 20 days post-inoculation. Red arrows represent the hyphal extension distance of each strain toward *S. sclerotiorum*.

Analysis of in vitro activity against the pathogenic fungus *S. sclerotiorum* showed that the hyphal extension abilities of ΔCrGlu6 mutants were markedly lower than those of the wild type strain (33.69%, *p* < 0.05) after co-culturing for 20 days. The complemented strains ΔCrGlu6-C recovered the antagonistic activities almost to the wild type levels (3.19%, Figure 7).

### 3.6. Characterization of CrGlu6 in Mycoparasitism

**Figure 8:**
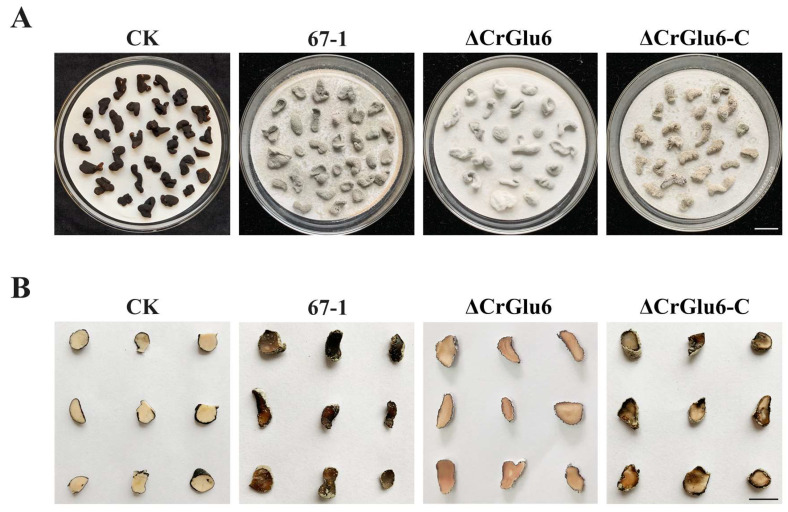
Mycoparasitism of *C. chloroleuca* strains against *S. sclerotiorum* sclerotia. (**A**) External phenotypes of healthy and infected sclerotia. Bar = 1 cm. (**B**) Transection of infected and uninfected sclerotia. Pictures were taken after 7 days of incubation at 26 °C. Bar = 0.5 cm.

After 7 days of cultivation in moisture, the mycoparasitic ability of ΔCrGlu6 against *S. sclerotiorum* sclerotia was significantly reduced compared with the wild type 67-1 and complemented strains ΔCrGlu6-C. From the external phenotypes and inner structures of the sclerotia, we could see that the sclerotia infected by the wild type strain were completely softened and rotten, resulting in the highest parasitic severity of grade 3. By comparison, though the surfaces of the sclerotia treated with *CrGlu6*-deficient mutants were covered with the hyphae of the biocontrol fungus, no soft and browned texture was apparent, which was equivalent to parasitic grade 2. When *CrGlu6* was complemented, the mycoparasitic ability was recovered (Figure 8), indicating that the *CrGlu6* gene is involved in mycoparasitism of *C. chloroleuca*.

### 3.7. Characterization of CrGlu6 in Control Efficacy

**Figure 9:**
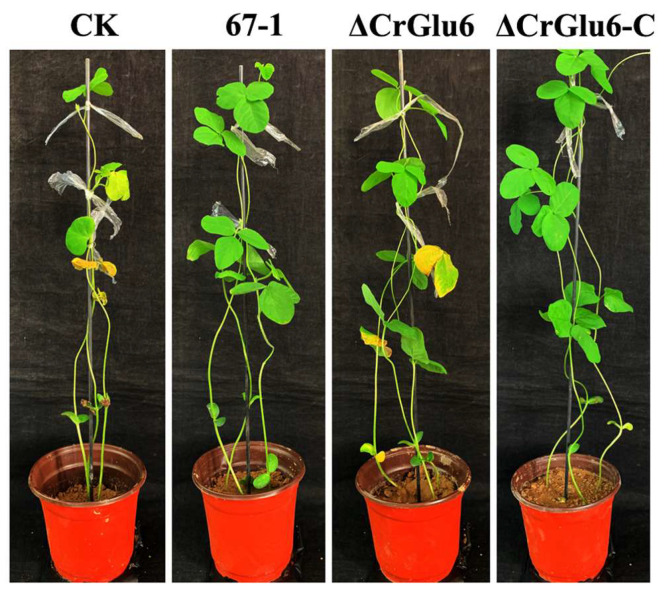
Control of sclerotinia stem rot of soybean by 67-1, ΔCrGlu6, and ΔCrGlu6-C. Plants treated with sterile water followed by the *S. sclerotiorum* pathogen served as controls (CK), and 15 pots were examined for each strain. The disease severity of sclerotinia stem rot was calculated after 7 days.

After inoculation with *S. sclerotiorum* for 7 days, severe leaf lesions were observed in the control soybean seedlings. However, seedlings treated with strain 67-1 presented less damage, achieving excellent control efficacy against sclerotinia stem rot (68.2%). When the *CrGlu6* gene was deleted, the control efficacy of the mutants decreased sharply (26.4%, *p* < 0.05) compared to the wild type strain, and the efficacy was regained in the complemented strains (Figure 9, Table 1), demonstrating that *CrGlu6* could dramatically affect the biocontrol efficacy of *C. chloroleuca*.

## 4. Discussion

MAPK signaling pathways have been found in all eukaryotes and play important roles in cell growth, differentiation, and stress responses [27,58,59]. Though an excellent roadmap was drawn for the model organism *S. cerevisiae*, illuminating MAPK signaling cascades, functional analyses in individual pathogenic and biocontrol fungi are required to comprehend the intricate roles of these signaling components in specific pathogen–host interactions [27,28]. During interactions, MAPK pathways are initiated once fungal cells perceive external stimuli, triggering cellular responses [31,60]. In a previous study, we confirmed that the MAPK enzyme Crmapk acts as a central regulator of signal transduction pathways, and it regulates the mycoparasitic ability and biocontrol efficacy of *C. chloroleuca* [35]. However, the regulatory mechanisms remain unclear.

Signal transduction relies on specific protein–protein interactions. In MAPK pathways, MAPKs phosphorylate substrates, mainly transcription factors, thereby regulate diverse biological processes [30,61]. In some filamentous fungi, such as *Aspergillus nidulans* and *F. oxysporum*, MAPKs function alongside the transcription factor Ste12 to initiate the production of secondary metabolites and to control hyphal growth and virulence [37,62]. Other kinds of receptors act downstream of MAPKs to participate in various pathways. In *M. grisea*, MAPKs cooperating with the heat shock factor Sfl1 significantly reduce virulence against rice and barley [27]. Moreover, a lack of MAPK phosphatase Msg5 in *S. cerevisiae* prevents the activation of gene transcription and protein phosphorylation controlled by MAPK Kss1 [63]. In our study on *C. chloroleuca*, several proteins involved in gene regulation, metabolism, and signal transduction processes were found to interact with Crmapk [38]. In the present work, the interaction between glucose-6-phosphate 1-epimerase CrGlu6 and Crmapk was further confirmed via multiple protein–protein interaction assays, including Y2H, GST pull-down, and BiFC techniques, indicating that CrGlu6 might associate with the MAPK pathway to influence mycoparasitism of *C. chloroleuca*.

In an organism, signal transduction occurs following stimulation to regulate cellular processes, including glucose homeostasis [44]. Upon activation of a pathway, protein kinases determine the output of metabolic processes via transcriptional and post-translational regulation of key enzymes mainly correlated with cellular metabolism, such as glucose-6-phosphate 1-epimerase [44,64]. Cellular metabolism defines the energetic status of cells and governs all stages of fungal growth and development, through various basic cellular building blocks such as lipids, amino acids, carbohydrates, nucleotides, and numerous enzymes and cofactors [65]. In the present study, we found that the glucose-6-phosphate 1-epimerase CrGlu6 interacts with the MAPK Crmapk in *C. chloroleuca*. A deficiency of *CrGlu6* induced the production of glycogen that plays a crucial role in energy metabolism and is generally considered the first choice for energy storage and utilization in cells [66,67]. This suggests that the *CrGlu6* gene is involved in hyphal development and other biological activities of *C. chloroleuca* through glucose metabolism.

Mycoparasitism is the leading mechanism of the biocontrol of *C. chloroleuca*; when confronting a fungal host, the mycoparasite initiates the expression of genes associated with recognition, penetration, and parasitism [17]. We found that the expression of *CrGlu6* was significantly upregulated during *C. chloroleuca* colonizing *S. sclerotiorum* sclerotia, implying that the *CrGlu6* gene might be indispensable for mycoparasitism of the biocontrol fungus. To achieve effective infection, high densities of fungal biomass and conidia are usually required. Our results showed that disruption of *CrGlu6* in *C. chloroleuca* markedly lowered conidiation but did not affect the mycelial growth rate; consequently, there was decreased invasion to the pathogen. Various factors including the reduced rates of conidial germination, abnormal cellular morphology, and mycelial branches might lead to these results, which need additional studies. Antagonistic ability refers to the control action of *C. chloroleuca* against pathogenic fungi through multiple mechanisms, including nutrient competition and direct mycoparasitism, which involves the production of antifungal metabolites and cell-wall degrading enzymes [68]. In our study, the hyphae of *C. chloroleuca* overgrew *S. sclerotiorum* when the fungi were co-cultured. By contrast, antifungal activities were greatly reduced for *CrGlu6*-deficient mutants, and control efficacy against soybean rot was significantly decreased in the greenhouse. Research on the direct interaction of the mycoparasite and its prey, including recognition, enwind, penetration, and disintegration will be very interesting [69]. Research on these phenotypes is underway to explore the mechanisms connecting the *CrGlu6* gene to the vegetative growth and biocontrol activities of *C. chloroleuca*.

Although there are several reports on the functions of aldose 1-epimerases in fungi [40,70], interactions between aldose 1-epimerase and MAPK pathways are not clear. Our current results confirmed that the glucose-6-phosphate 1-epimerase CrGlu6 interacts with Crmapk, and this interaction modulates the mycoparasitic action of *C. chloroleuca*. We speculate that CrGlu6 is induced once *C. chloroleuca* encounters its mycohosts, and physiological processes such as growth and conidiation are subsequently affected, reducing mycoparasitic ability and biocontrol efficacy of the fungus via MAPK pathways regulated by Crmapk. To the best of our knowledge, this is the first report of an interaction between a MAPK and aldose 1-epimerase in biocontrol fungi. The findings provide new insight into the mechanisms by which glucose-6-phosphate 1-epimerase regulates mycoparasitism of *C. chloroleuca*. In future work, other metabolic pathways and functional analyses will be conducted to elucidate the regulatory mechanisms of MAPKs and their interacting proteins in mycoparasitism of *C. chloroleuca*.

## 5. Conclusions

By bioinformatics and protein–protein interaction analyses, glucose-6-phosphate 1-epimerase CrGlu6 was verified to interact with Crmapk during *C. chloroleuca* parasitizing *S. sclerotiorum*. The CrGlu6 protein was essential for fungal growth, conidiation, and biocontrol activities, indicating that it might be involved in the mycoparasitism of *C. chloroleuca* under MAPK-mediated regulation. The findings illuminate the functions and regulation of aldose 1-epimerase in *C. chloroleuca* and help to further reveal the regulatory mechanisms of MAPKs and their interacting proteins underlying mycoparasitism.

## Figures and Tables

**Table 1:** Control efficacies of *C. chloroleuca* strains against sclerotinia stem rot.

Strain	Disease Index	Control Efficacy (%)
CK	57.9 ± 1.1 a	-
67-1	18.4 ± 0.5 c	68.2 ± 0.8 a
ΔCrGlu6	42.6 ± 1.3 b	26.4 ± 1.2 b
ΔCrGlu6-C	19.2 ± 0.4 c	66.8 ± 0.9 a

Data are means ± standard deviations of three replicates for the three mutants. Different letters in a column indicate significant differences according to Tukey’s test (*p* < 0.05).

## Data Availability

The data that support the findings of this study are available from the corresponding author upon reasonable request.

## Supplementary Materials

The following are available online at https://www.mdpi.com/article/10.3390/jof9070764/s1 Figure S1: Impact of CrGlu6 deficiency on mycelial growth. Mycelial growth of 67-1, ΔCrGlu6, and ΔCrGlu6-C on PDA medium after 3, 6, and 9 days of incubation. The data are the means of three mutants, and the means and standard errors are calculated from three independent repeats. Statistical tests were performed by Tukey test for multiple comparisons. Table S1: Primers used in this study.

## References

[1] Xue A.G. (2003). Biological Control of Pathogens Causing Root Rot Complex in Field Pea Using *Clonostachys rosea* Strain ACM941. Phytopathology.

[2] Zhang L., Yang J., Niu Q., Zhao X., Ye F., Liang L., Zhang K.-Q. (2008). Investigation on the infection mechanism of the fungus *Clonostachys rosea* against nematodes using the green fluorescent protein. Appl. Microbiol. Biotechnol..

[3] Iqbal M., Dubey M., Broberg A., Viketoft M., Jensen D.F., Karlsson M. (2019). Deletion of the Nonribosomal Peptide Synthetase Gene *nps1* in the Fungus *Clonostachys rosea* Attenuates Antagonism and Biocontrol of Plant Pathogenic *Fusarium* and Nematodes. Phytopathology.

[4] Sun Z.-B., Sun M.-H., Li S.-D. (2015). Identification of mycoparasitism-related genes in *Clonostachys rosea* 67-1 active against *Sclerotinia sclerotiorum*. Sci. Rep..

[5] Iqbal M., Broberg M., Haarith D., Broberg A., Bushley K.E., Durling M.B., Viketoft M., Jensen D.F., Dubey M., Karlsson M. (2020). Natural variation of root lesion nematode antagonism in the biocontrol fungus *Clonostachys rosea* and identification of biocontrol factors through genome-wide association mapping. Evol. Appl..

[6] Keyser C.A., Jensen B., Meyling N.V. (2016). Dual effects of *Metarhizium* spp. and *Clonostachys rosea* against an insect and a seed-borne pathogen in wheat. Pest Manag. Sci..

[7] Toledo A., Virla E., Humber R., Paradell S., Lastra C.L. (2006). First record of *Clonostachys rosea* (Ascomycota: Hypocreales) as an entomopathogenic fungus of *Oncometopia tucumana* and *Sonesimia grossa* (Hemiptera: Cicadellidae) in Argentina. J. Invertebr. Pathol..

[8] Jiang C.-X., Yu B., Miao Y.-M., Ren H., Xu Q., Zhao C., Tian L.-L., Yu Z.-Q., Zhou P.-P., Wang X. (2021). Indole Alkaloids from a Soil-Derived *Clonostachys rosea*. J. Nat. Prod..

[9] Tzelepis G., Karlsson M., Jensen D.F., Dubey M. (2015). Identifying glycoside hydrolase family 18 genes in the mycoparasitic fungal species *Clonostachys rosea*. Microbiology.

[10] Atanasova L., Dubey M., Grujic M., Gudmundsson M., Lorenz C., Sandgren M., Kubicek C.P., Jensen D.F., Karlsson M. (2018). Evolution and functional characterization of pectate lyase PEL12, a member of a highly expanded *Clonostachys rosea* polysac-charide lyase 1 family. BMC Microbiol..

[11] Sun Z.-B., Wang Q., Sun M.-H., Li S.-D. (2019). The heat shock protein 70 gene is involved for colony morphology, sporulation and mycoparasitism of *Clonostachys rosea*. FEMS Microbiol. Lett..

[12] Moreira G.M., Abreu L.M., Carvalho V.G., Schroers H.J., Pfenning L.H. (2016). Multilocus phylogeny of *Clonostachys subgenus* Bi-onectria from Brazil and description of *Clonostachys chloroleuca* sp. nov. Mycol. Prog..

[13] Abreu L.M., Moreira G.M., Ferreira D., Rodrigues-Filho E., Pfenning L.H. (2014). Diversity of *Clonostachys* species assessed by mo-lecular phylogenetics and MALDI-TOF mass spectrometry. Fungal Biol..

[14] Tian T., Li S.-D., Sun M.-H. (2014). Synergistic Effect of Dazomet Soil Fumigation and *Clonostachys rosea* against Cucumber Fusarium Wilt. Phytopathology.

[15] Sun Z.-B., Sun M.-H., Li S.-D. (2015). Draft Genome Sequence of Mycoparasite *Clonostachys rosea* Strain 67-1. Genome Announc..

[16] Silva H.A.O., Teixeira W.D., Borges Á.V., Junior A.L.S., Alves K.S., Junior O.M.R., Abreu L.M. (2021). Biocontrol of potato early blight and suppression of *Alternaria grandis* sporulation by *Clonostachys* spp. Plant Pathol..

[17] Karlsson M., Atanasova L., Jensen D.F., Zeilinger S. (2017). Necrotrophic Mycoparasites and Their Genomes. Microbiol. Spectr..

[18] Alfiky A., Weisskopf L. (2021). Deciphering *Trichoderma*–Plant–Pathogen Interactions for Better Development of Biocontrol Applications. J. Fungi.

[19] Herrera C.S., Hirooka Y., Chaverri P. (2016). Pseudocospeciation of the mycoparasite *Cosmospora* with their fungal hosts. Ecol. Evol..

[20] Guzmán-Guzmán P., Alemán-Duarte M.I., Delaye L., Herrera-Estrella A., Olmedo-Monfil V. (2017). Identification of effector-like proteins in *Trichoderma* spp. and role of a hydrophobin in the plant-fungus interaction and mycoparasitism. BMC Genet..

[21] Martínez-Soto D., Ruiz-Herrera J. (2017). Functional analysis of the MAPK pathways in fungi. Rev. Iberoam. Micol..

[22] Hong Y., Liu Q., Cao Y., Zhang Y., Chen D., Lou X., Cheng S., Cao L. (2019). The OsMPK15 Negatively Regulates Magnaporthe oryza and Xoo Disease Resistance via SA and JA Signaling Pathway in Rice. Front. Plant Sci..

[23] Li T., Xiu Q., Wang J., Duan Y., Zhou M. (2021). A putative MAPK kinase kinase gene Ssos4 is involved in mycelial growth, virulence, osmotic adaptation, and sensitivity to fludioxonil and is essential for SsHog1 phosphorylation in *Sclerotinia sclerotiorum*. Phyto-pathology.

[24] González-Rubio G., Fernández-Acero T., Martín H., Molina M. (2019). Mitogen-Activated Protein Kinase Phosphatases (MKPs) in Fungal Signaling: Conservation, Function, and Regulation. Int. J. Mol. Sci..

[25] Krysan P.J., Colcombet J. (2018). Cellular Complexity in MAPK Signaling in Plants: Questions and Emerging Tools to Answer Them. Front. Plant Sci..

[26] Rispail N., Soanes D.M., Ant C., Czajkowski R., Grünler A., Huguet R., Perez-Nadales E., Poli A., Sartorel E., Valiante V. (2009). Comparative genomics of MAP kinase and calcium-calcineurin signalling components in plant and human pathogenic fungi. Fungal Genet. Biol..

[27] Hamel L.-P., Nicole M.-C., Duplessis S., Ellis B.E. (2012). Mitogen-Activated Protein Kinase Signaling in Plant-Interacting Fungi: Distinct Messages from Conserved Messengers. Plant Cell.

[28] Leng Y., Zhong S. (2015). The Role of Mitogen-Activated Protein (MAP) Kinase Signaling Components in the Fungal Development, Stress Response and Virulence of the Fungal Cereal Pathogen *Bipolaris sorokiniana*. PLoS ONE.

[29] Choudhury S., Baradaran-Mashinchi P., Torres M.P. (2018). Negative feedback phosphorylation of gamma subunit Ste18 and the Ste5 scaffold synergistically regulates MAPK activation in yeast. Cell Rep..

[30] Jiang C., Zhang X., Liu H., Xu J.-R. (2018). Mitogen-activated protein kinase signaling in plant pathogenic fungi. PLoS Pathog..

[31] Zhang X., Wang Z., Jiang C., Xu J.-R. (2021). Regulation of biotic interactions and responses to abiotic stresses by MAP kinase pathways in plant pathogenic fungi. Stress Biol..

[32] Gruber S., Zeilinger S. (2014). The Transcription Factor Ste12 Mediates the Regulatory Role of the Tmk1 MAP Kinase in Mycoparasitism and Vegetative Hyphal Fusion in the Filamentous Fungus *Trichoderma atroviride*. PLoS ONE.

[33] Mendoza-Mendoza A., Rosales-Saavedra T., Cortés C., Castellanos-Juárez V., Martínez P., Herrera-Estrella A. (2007). The MAP kinase TVK1 regulates conidiation, hydrophobicity and the expression of genes encoding cell wall proteins in the fungus *Trichoderma virens*. Microbiology.

[34] Zeng F., Gong X., Hamid M.I., Fu Y., Jiatao X., Cheng J., Li G., Jiang D. (2012). A fungal cell wall integrity-associated MAP kinase cascade in *Coniothyrium minitans* is required for conidiation and mycoparasitism. Fungal Genet. Biol..

[35] Sun Z.-B., Wang Q., Sun M.-H., Li S.-D. (2020). The Mitogen-Activated Protein Kinase Gene *Crmapk* Is Involved in *Clonostachys chloroleuca* Mycoparasitism. Mol. Plant-Microbe Interact..

[36] Park G., Xue C., Zheng L., Lam S., Xu J.-R. (2002). *MST12* Regulates Infectious Growth but not Appressorium Formation in the Rice Blast Fungus *Magnaporthe grisea*. Mol. Plant-Microbe Interact..

[37] Rispail N., Di Pietro A. (2009). *Fusarium oxysporum* Ste12 Controls Invasive Growth and Virulence Downstream of the Fmk1 MAPK Cascade. Mol. Plant-Microbe Interact..

[38] Lv B., Fan L., Li S., Sun M. (2022). Screening and characterisation of proteins interacting with the mitogen-activated protein kinase Crmapk in the fungus *Clonostachys chloroleuca*. Sci. Rep..

[39] Li S., Ha S.-J., Kim H.J., Galazka J.M., Cate J.H., Jin Y.-S., Zhao H. (2013). Investigation of the functional role of aldose 1-epimerase in engineered cellobiose utilization. J. Biotechnol..

[40] Samolski I., de Luis A., Vizcaíno J.A., Monte E., Suárez M.B. (2009). Gene expression analysis of the biocontrol fungus *Trichoderma harzianum* in the presence of tomato plants, chitin, or glucose using a high-density oligonucleotide microarray. BMC Microbiol..

[41] Gowda M., Venu R.C., Raghupathy M.B., Nobuta K., Malali G., Wing R., Stahlberg E., Couglan S., Haudenschild C.D., Dean R. (2006). Deep and comparative analysis of the mycelium and appressorium transcriptomes of *Magnaporthe grisea* using MPSS, RL-SAGE, and oligoarray methods. BMC Genom..

[42] Seiboth B., Karaffa L., Sandor E., Kubicek C. (2002). The *Hypocrea jecorina* gal10 (uridine 5′-diphosphate-glucose 4-epimerase-encoding) gene differs from yeast homologues in structure, genomic organization and expression. Gene.

[43] Chance E.M., Hess B., Plesser T., Wurster B. (1975). Determination of the kinetic constants of glucose-6-phosphate l-epimerase by non-linear optimization. Eur. J. Biochem..

[44] Schultze S.M., Hemmings B.A., Niessen M., Tschopp O. (2012). PI3K/AKT, MAPK and AMPK signalling: Protein kinases in glucose homeostasis. Expert Rev. Mol. Med..

[45] Rodríguez M., Cabrera G., Gozzo F., Eberlin M., Godeas A. (2011). *Clonostachys rosea* BAFC3874 as a *Sclerotinia sclerotiorum* antagonist: Mechanisms involved and potential as a biocontrol agent. J. Appl. Microbiol..

[46] Zhang Y.H., Gao H.L., Ma G.Z., Li S.D. (2004). Mycoparasitism of *Gliocladium roseum* 67-1 on *Sclerotinia sclerotiorum*. Acta Phytopathol. Sin..

[47] Tang G.F., Chen A.A., Dawood D.H., Liang J.T., Chen Y., Ma Z.H. (2020). Capping proteins regulate fungal development, DON-toxisome formation and virulence in *Fusarium graminearum*. Mol. Plant Pathol..

[48] Xu G., Zhong X., Shi Y., Liu Z., Jiang N., Liu J., Ding B., Li Z., Kang H., Ning Y. (2020). A fungal effector targets a heat shock-dynamin protein complex to modulate mitochondrial dynamics and reduce plant immunity. Sci. Adv..

[49] Larkin M.A., Blackshields G., Brown N.P., Chenna R., McGettigan P.A., McWilliam H., Valentin F., Wallace I.M., Wilm A., Lopez R. (2007). Clustal W and Clustal X version 2.0. Bioinformatics.

[50] Kumar S., Stecher G., Tamura K. (2016). MEGA7: Molecular evolutionary genetics analysis version 7.0 for bigger datasets. Mol. Biol. Evol..

[51] Livak K.J., Schmittgen T.D. (2001). Analysis of relative gene expression data using real-time quantitative PCR and the 2^−∆∆Ct^ method. Methods.

[52] Frandsen R.J., Andersson J.A., Kristensen M.B., Giese H. (2008). Efficient four fragment cloning for the construction of vectors for targeted gene replacement in filamentous fungi. BMC Mol. Biol..

[53] Kong X., Zhang H., Wang X., van der Lee T., Waalwijk C., van Diepeningen A., Brankovics B., Xu J., Xu J., Chen W. (2019). FgPex3, a Peroxisome Biogenesis Factor, Is Involved in Regulating Vegetative Growth, Conidiation, Sexual Development, and Virulence in *Fusarium graminearum*. Front. Microbiol..

[54] Sun Z.-B., Sun M.-H., Zhou M., Li S.-D. (2017). Transformation of the endochitinase gene Chi67-1 in *Clonostachys rosea* 67-1 increases its biocontrol activity against *Sclerotinia sclerotiorum*. AMB Express.

[55] Filizola P.R.B., Luna M.A.C., De Souza A.F., Coelho I.L., Laranjeira D., Campos-Takaki G.M. (2019). Biodiversity and phylogeny of novel *Trichoderma* isolates from mangrove sediments and potential of biocontrol against *Fusarium* strains. Microb. Cell Factories.

[56] Dubey M.K., Jensen D.F., Karlsson M. (2014). An ATP-Binding Cassette Pleiotropic Drug Transporter Protein Is Required for Xenobiotic Tolerance and Antagonism in the Fungal Biocontrol Agent *Clonostachys rosea*. Mol. Plant-Microbe Interact..

[57] Lv B., Jiang N., Hasan R., Chen Y., Sun M., Li S. (2020). Cell Wall Biogenesis Protein Phosphatase CrSsd1 Is Required for Conidiation, Cell Wall Integrity, and Mycoparasitism in *Clonostachys rosea*. Front. Microbiol..

[58] Wei W., Xiong Y., Zhu W., Wang N., Yang G., Peng F. (2016). *Colletotrichum higginsianum* Mitogen-Activated Protein Kinase ChMK1: Role in Growth, Cell Wall Integrity, Colony Melanization, and Pathogenicity. Front. Microbiol..

[59] Jagodzik P., Tajdel-Zielinska M., Ciesla A., Marczak M., Ludwikow A. (2018). Mitogen-Activated Protein Kinase Cascades in Plant Hormone Signaling. Front. Plant Sci..

[60] Tong S.-M., Feng M.-G. (2018). Insights into regulatory roles of MAPK-cascaded pathways in multiple stress responses and life cycles of insect and nematode mycopathogens. Appl. Microbiol. Biotechnol..

[61] Gao H., Jiang L., Du B., Ning B., Ding X., Zhang C., Song B., Liu S., Zhao M., Zhao Y. (2022). GmMKK4-activated GmMPK6 stimulates GmERF113 to trigger resistance to *Phytophthora sojae* in soybean. Plant J..

[62] Bayram O., Bayram S., Ahmed Y.L., Maruyama J.-I., Valerius O., Rizzoli S.O., Ficner R., Irniger S., Braus G.H. (2012). The Aspergillus nidulans MAPK Module AnSte11-Ste50-Ste7-Fus3 Controls Development and Secondary Metabolism. PLoS Genet..

[63] Marín M.J., Flández M., Bermejo C., Arroyo J., Martín H., Molina M. (2009). Different modulation of the outputs of yeast MAPK-mediated pathways by distinct stimuli and isoforms of the dual-specificity phosphatase Msg5. Mol. Genet. Genom..

[64] Luo B., Ma P., Nie Z., Zhang X., He X., Ding X., Feng X., Lu Q.X., Ren Z.Y., Lin H.J. (2019). Metabolite profiling and genome-wide association studies reveal response mechanisms of phosphorus deficiency in maize seedling. Plant J..

[65] Khan M., Djamei A. (2023). Co-immunoprecipitation-Based Identification of Effector-Host Protein Interactions from Pathogen-Infected Plant Tissue. Methods Mol. Biol..

[66] Roach P.J., Depaoli-Roach A.A., Hurley T.D., Tagliabracci V.S. (2012). Glycogen and its metabolism: Some new developments and old themes. Biochem. J..

[67] Keinan O., Valentine J.M., Xiao H., Mahata S.K., Reilly S.M., Abu-Odeh M., Deluca J.H., Dadpey B., Cho L., Pan A. (2021). *Glycogen metabolism* links glucose homeostasis to thermogenesis in adipocytes. Nature.

[68] Qualhato T.F., Lopes F.A.C., Steindorff A.S., Brandão R.S., Jesuino R.S.A., Ulhoa C.J. (2013). Mycoparasitism studies of *Trichoderma* species against three phytopathogenic fungi: Evaluation of antagonism and hydrolytic enzyme production. Biotechnol. Lett..

[69] Hasan R., Lv B., Uddin J., Chen Y., Fan L., Sun Z., Sun M., Li S. (2022). Monitoring Mycoparasitism of *Clonostachys rosea* against *Botrytis cinerea* Using GFP. J. Fungi.

[70] Kulcsár L., Flipphi M., Jónás Á., Sándor E., Fekete E., Karaffa L. (2017). Identification of a mutarotase gene involved in D-galactose utilization in *Aspergillus nidulans*. FEMS Microbiol. Lett..

