# Supplementary material for: Glucose-6-phosphate 1-Epimerase CrGlu6 Contributes to Development and Biocontrol Efficiency in Clonostachys chloroleuca
Source: J Fungi (Basel). 2023 Jul 20;9(7):764. doi: 10.3390/jof9070764 (PMC10381721; doi:10.3390/jof9070764)
Supplement: Supplementary file 1 [file jof-09-00764-s001.zip › jof-2490281-supplementary.pdf]

SUPPLEMENTARY DATA:

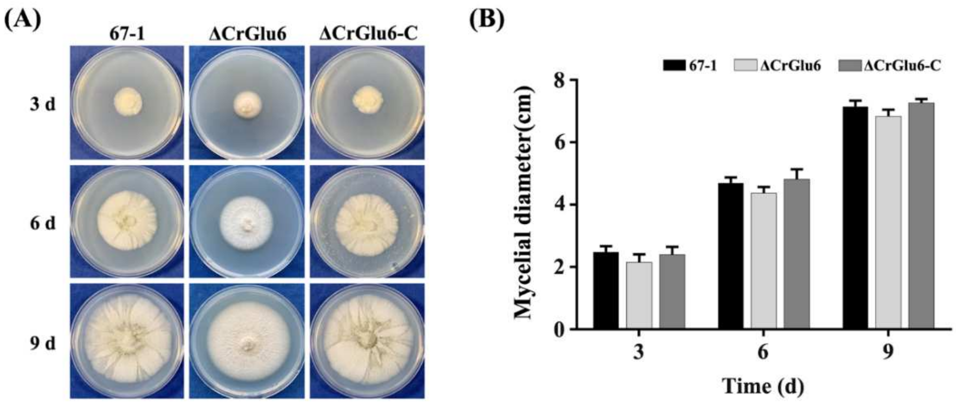

Figure S1. Impact of CrGlu6 deficiency on mycelial growth. Mycelial growth of 67-1, ΔCrGlu6, and ΔCrGlu6-C on PDA medium after 3, 6, and 9 days of incubation. The data are the means of three mutants, and the means and standard errors are calculated from three independent repeats. Statistical tests were performed by Tukey test for multiple comparisons.

Table S1 Primers used in this study

Primer	Sequence (5′–3′)	Relevant characteristics
CrGlu6-UF	GGTCTTAAU TGCAGGTCGTCATGATCACT	Amplification of the *CrGlu6* 5′ region for construction of *CrGlu6* deletion mutants
CrGlu6-UR	GGCATTAAU ACAAGTCCCTTTTCCTGCCA	
CrGlu6-DF	GGACTTAAU GGGTGGGTTAGGGAGATTGT	Amplification of the *CrGlu6* 3′ region for construction of *CrGlu6* deletion mutants
CrGlu6-DR	GGGTTTAAU CACCCTCATCACCCGCTAC	
Hph-F	TGGAGCTAGTGGAGGTCAACA	Amplification of the hygromycin resistant gene *hph*
Hph-R	CGGTCGGCATCTACTCTATTC	
CrGlu6-In-F	TACCAATCCGCATCGCATTG	Identification of *CrGlu6* deletion transformants
CrGlu6-In-R	ATTCAATGCGACCAGAGAGC	
CrGlu6-Yz-F	CTCATGCTGTTCGGTTCCAT	Identification of *CrGlu6* deletion transformants
CrGlu6-Yz-R	AGAACTCCTCCGTGCCCG	
CrGlu6-COMF	CCCCCGGGCTGCAGGAATTCAT GCTGTTCGGTTCCATTGG	*CrGlu6* complementation

CrGlu6-COMR	TCGACGGTATCGATAAGCTTCTT GAGTTGGCGTTCTTGGT	
CrGlu6-F	GCTCGTCTTCCCGGTTTTC	Identification of *CrGlu6* expression levels in qRT-PCR and RT-PCR assays
CrGlu6-R	CTCAAAGGAAGTCTCGCCGT	
EF1-F	TCGATGTCGCTCCTGACT	Amplification of the reference gene *EF1* in qRT-PCR and RT-PCR assays
EF1-R	AGCGTGACCGTTTATTTGA	
Crmapk-BD-F	CATGGAGGCCGAATTCATGTCT CGATCAACTCAGCCCAGC	Construction of pGBKT7-Crmapk for Y2H assay
Crmapk-BD-R	GCAGGTCGACGGATCCTCATCG CATGACCTCCTGGTAGAT	
CrGlu6-AD-F	GGAGGCCAGTGAATTCATGGCC ACTCTCGCCTCTTGGACG	Construction of pGADT7-CrGlu6 for Y2H assay
CrGlu6-AD-R	CGAGCTCGATGGATCCCTAGGG GAGGGAGATGGTCTGGGC	
Crmapk-65-F	CAGATCTTGGCTTTCGTAGGAA CCCAATCTTCAATGTCTCGATCA ACTCAGCC	Construction of YFP^N-Crmapk for BiFC assay
Crmapk-65-R	GCTCACCATCGTGGCGATGGAG CGTCGCATGACCTCCTGGTAGA	
CrGlu6-68-F	CAGATCTTGGCTTTCGTAGGAA CCCAATCTTCAATGGCCACTCTC GCCTCTTG	Construction of CrGlu6-YFP^C for BiFC assay
CrGlu6-68-R	GTTCGGGATCTTGCAGGCCGGG CGGGGGAGGGAGATGGTCTGG GC	
